# Influence of Rest on Players’ Performance and Physiological Responses during Basketball Play

**DOI:** 10.3390/sports5020027

**Published:** 2017-05-10

**Authors:** Robert G. Crowther, Anthony S. Leicht, Jessica M. Pohlmann, Jane Shakespear-Druery

**Affiliations:** 1 School of Health Sciences, University of South Australia, Adelaide, 5001 Australia; 2 Sport and Exercise Science, James Cook University, Townsville, 4811, Australia; Anthony.Leicht@jcu.edu.au; 3 Sport and Exercise, University of Southern Queensland, Ipswich 4305, Australia; Jessica.Pohlmann@outlook.com (J.M.P.); Jane.Shakespear-Druery@usq.edu.au (J.S.-D.)

**Keywords:** team sport, core temperature, skin temperature, countermovement jump, substitution player, performance

## Abstract

Pre-match warm-ups are standard in many sports but the focus has excluded the substitute players. The aim of this research was to investigate the result of inactivity on physiological and performance responses in substitute basketball players during competition. Two basketball players from the second tier of the State League of Queensland, Australia volunteered for this study and were assessed for performance (countermovement jump—CMJ) and physiological (core temperature via ingestible pill; skin temperature at the arm, chest, calf and thigh; heart rate—HR) responses prior to and following a 20-min warm-up, and during the first half of a competitive basketball match (2 × 20-min real time quarters). Warm up resulted in increases in CMJ (~7%), HR (~100 bpm) and core (~0.8 °C) and skin (~1.0 °C) temperatures. Following the warm up and during inactivity, substitute players exhibited a decrease in all responses including CMJ (~13%), HR (~100 bpm), and core (~0.5 °C) and skin (~2.0 °C) temperatures. Rest resulted in reductions in key performance and physiological responses during a competitive match that poses a risk for match strategies. Coaches should consider implementing a warm up to enhance core/skin temperature for substitute players immediately before they engage with competition to optimise player performance.

## 1. Introduction

Pre-match warm-ups have been standard practice for athletes in many sports with the intent to prepare the athlete for competition [1]. This is typically achieved using dynamic warm-up exercises such as interval and shuttle running, cycle ergometers, etc. to enhance muscle and/or body temperature [1,2]. While this preparation provides significant stimulus for the athletes prior to the commencement of competition, there are other athletes within a team that remain inactive following the pre-match warm up—the substitutes. In recent years, sport scientists have focused on increasing substitute player readiness in many team sports such as soccer and rugby [3,4]. However, such a focus has been limited for basketball. Recently Gomez et al. [5] reported that scoring performance was significantly and positively enhanced, particularly in the first quarter, immediately following the substitution of a player during a competitive basketball match. This result was remarkable given that substitute players typically engage in a match following a substantial period of inactivity. Basketball substitutes can remain inactive for up to >30 min before they engage in a match and do not undertake any activity before match play, unlike other team sport athletes. Given that basketball can involve significant increases in core body temperature during the game and potential muscle damage as indicated by greater creatine-kinase levels 24 and 48 h post game [6] it would seem advantageous to make sure a substitute player wasn’t further negatively affected due to inactivity prior to entering the game. Previously, Galazoulas et al. [7] reported a decline in performance for basketball players who remained inactive following a warm-up. However, this study did not examine responses during real competition with official matches reported to generate greater stress compared to simulated conditions [8].

Therefore, the aim of this preliminary study was to investigate the result of inactivity or rest on physiological responses such as core temperature, skin temperature, heart rate and vertical jump performance in substitute basketball players during official competition. We focused on core and skin temperatures as performance changes with warm up have been credited to temperature-related mechanisms [1]. A greater understanding of the impact of inactivity on basketball players’ performance and physiological responses would provide the foundation for player management to optimize team performance and match outcome.

## 2. Methods

### 2.1. Subjects

Two basketball players (Player 1–20 years, 190.0 cm, 96.9 N, 17.4% body fat; Player 2–19 years, 196.0 cm, 85.2 N, 11.0% body fat) were recruited from the second tier of the State Basketball League of Queensland, Australia to participate in this study. Subjects performance (countermovement jump) and physiological (core and skin temperature, heart rate (HR)) responses were examined prior to (pre-warm up) and following a 20-min warm-up (post-warm up), and during the first half of a competitive basketball match (i.e., 2 × 20-min quarters). The first 2 quarters of a match were chosen as this reflected the maximal period of inactivity for players during a match with players able to undertake a warm up (5–10 min) before the start of the 3rd quarter. Further, to maximize the applicability of the study results, players were engaged with the match and expecting to participate at any moment (i.e., they were not informed that they would be resting during the match). The environmental conditions remained constant throughout the warm-up and match half (19.95–19.58 °C; 54.34–55.33% relative humidity). Players wore only playing clothes, no jacket or towel over legs during the study were allowed to eliminate any effect of extra clothing. All protocols and assessments were conducted in accordance with the Declaration of Helsinki and were approved by the institution Human Research Ethics Committee.

### 2.2. Procedures

#### Warm-Up

Each subject engaged in the team warm-up that consisted of dynamic running actions (i.e., A-skip, B-skip, lunges, squats, leg swings, open/close gate, 2 min of half court sprints) and basketball specific actions (i.e., lay-ups, jump shot, 3-point shot, catching, passing and offensive decision making involving catching the ball and choosing to either shoot or drive).

### 2.3. Measures

#### 2.3.1. Anthropometric Assessment

Subject’s height, weight and percentage body fat were measured before the match warm-up. Height was assessed using a portable telescopic plastic stadiometer (Marsden Leicester Height Measure, Marsden Weighing Group, Rotherham, UK). Body weight and percentage body fat were measured using bioelectrical impedance scales (TANITA BC541 Digital scale, TANITA, Kewdale, WA, Australia).

#### 2.3.2. Counter Movement Jump

Each subject performed three stationary double-leg counter-movement jumps (CMJ) to assess jumping performance, prior to and immediately following the warm-up and following the match half. Each CMJ was conducted using a Swift Yardstick (Swift Performance, Wacol, Australia) positioned beside the subjects dominate arm side (right for both subjects), with the best result at each time point analysed.

#### 2.3.3. Core Temperature

Subjects swallowed a core temperature wireless sensor (CorTemp^®^, HQ Inc., Palmetto, FL, USA) at least 6 h before warm-up. Body core temperature (TCORE) was recorded using the wireless monitoring data recorder (CorTemp Data Recorder, HQ Inc., Palmetto, FL, USA) at pre-warm up, post-warm up and every minute during the competitive match.

#### 2.3.4. Skin Temperature

Four surface temperature buttons (Thermochron iButtons, Maxim Integrated Products, San Jose, CA, USA), sampling at 1 Hz, were affixed by breathable strapping tape at the following prepared sites of each subject: forearm (midpoint between the radial and ulna head on the posterior surface of the arm, TARM), chest (half-way between the axillary fold and the manubrium, most superior point on the sternum, TCHEST), thigh (midpoint between the inguinal fold and superior border of the patella, midpoint transversely across the leg, TTHIGH), and calf (widest circumference of posterior surface in line with the Achilles tendon, TCALF). Body mean skin temperature (TSK) was calculated using the following Equation [9].

TSK = 0.2 × (THIGH+ CALF) + 0.3 (CHEST + ARM)

#### 2.3.5. Heart Rate

Each subject wore a Polar Team HR monitor (Polar Electro, Smeaton Grange, Australia), sampling at a rate of 1 Hz and was recorded at the start of each minute throughout the warm up and match half.

## 3. Results

### 3.1. CMJ Performance

At post-warm up, both subjects displayed an improvement in CMJ performance from pre-warm up values (Player 1: 72 cm to 78 cm, 8.3% improvement; Player 2: 79 cm to 84 cm, 6.0% improvement). Following the match half, both subjects exhibited a reduced CMJ performance compared to post warm-up (Player 1: 78 cm vs. 66 cm, 15.4% drop; Player 2: 84 cm vs. 74 cm, 11.9% drop) with these results also lower than pre-warm up values (~7%).

### 3.2. Core Temperature

**Figure 1:**
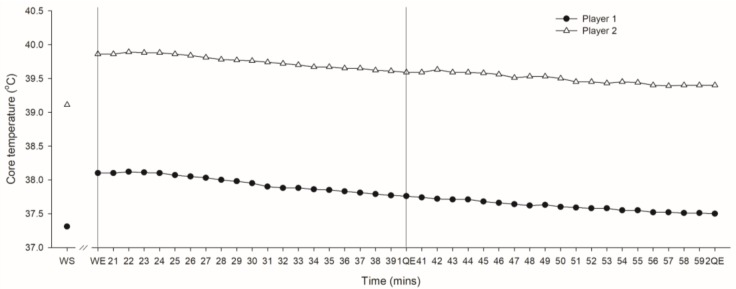
Core temperature for Player 1 and 2 at the start (WS) and end (WE) of warm up and throughout the first (21-1QE) and second (41-2QE) quarters of play. QE—quarter end.

Both subjects’ TCORE increased during the warm-up and then continued to steadily reduce over the course of the match half (~0.5 °C) with values remaining higher than pre warm-up levels (see Figure 1).

### 3.3. Skin Temperature

**Figure 2:**
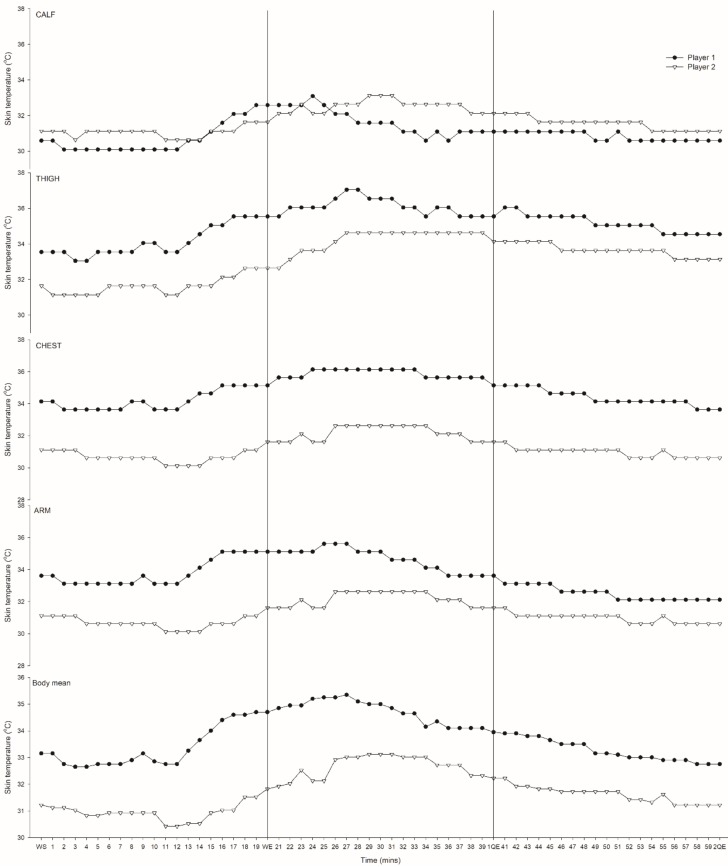
Calf, thigh, chest, arm and body mean skin temperature for Player 1 and 2 at the start (WS) and end (WE) of warm up and throughout the first (21-1QE) and second (41-2QE) quarters of play. QE—quarter end.

All monitored areas (calf, thigh, chest and arm) exhibited increases in temperature during the warm-up with varying post warm-up responses. For both players, TCALF peaked at 4–11 min, TTHIGH peaked at 8–20 min, TCHEST peaked at 13–14 min, and TARM peaked at 7–14 min, post-warm up followed by a decline (~1–2 °C) (Figure 2). Only TTHIGH values remained higher than pre-warm up temperatures throughout the match half. Overall, TSK peaked at ~6 min for Player 1 and ~11 min for Player 2 with a steady decline (~2 °C) thereafter (see Figure 2).

### 3.4. Heart Rate

**Figure 3:**
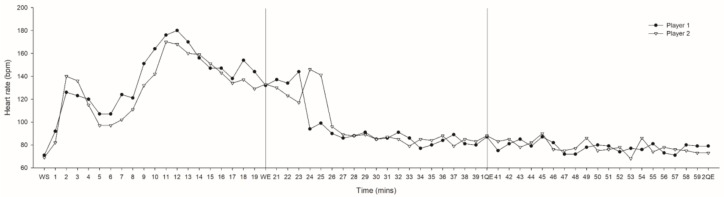
Heart rate for Player 1 and 2 at the start (WS) and end (WE) of warm up and throughout the first (21-1QE) and second (41-2QE) quarters of play. QE—quarter end.

During the warm-up, Player 1’s HR increased and peaked at 180 bpm (12 min) with a 102.8% change between start and end of warm-up. Player 2’s HR increased and peaked at 170 bpm (11 min) with an 87.0% change between start and end of warm-up. By the 6th minute of the match half, HR dropped to 90 bpm and 96 bpm for Player 1 and 2 respectively and thereafter continued to slowly decrease over the course of the remaining time (see Figure 3). Across the first quarter there was a 37.4% and 37.6% decrease in HR for Player 1 and 2 respectively. Across the second quarter there was a 5.3% increase for Player 1 and 12% decrease for Player 2.

## 4. Discussion

The aim of this brief report was to investigate the effect of match inactivity on basketball players following a typical warm-up and not engaging with the match before half-time. Extended rest on the sideline resulted in reductions in CMJ performance, HR, TCORE and skin temperatures (particularly at the arm and calf) during the competitive match. Substitute players therefore may not be optimally prepared physiologically for basketball competition. Coaches should consider implementing additional activities for substitute players prior to match engagement.

Recently Gomez et al. [5] reported that scoring performance during a competitive basketball match was positively enhanced immediately following the substitution of a player. This strategic action highlights an important stage of competition that may impact on overall match outcome. However, the current results indicated that the substitutes may in fact pose a risk to match outcome by being physiologically underprepared in comparison to a standard, pre-match warm up. The benefits of warm up have been discussed with most attributed to temperature-related mechanisms [1]. Our current result highlight an important and continual reduction in both TCORE and TSK with inactivity for substitutes, particularly with greater rest time. During a simulated warm-up and rest period, Galazoulas et al. [7] reported that aural temperature was decreased by ~0.8 °C over a 40-min period. This decrease was slightly greater than the TCORE result in the current study with differences in core temperature recording methods likely for the disparity. Regardless, the substantial reduction in TCORE by athletes in the current study was accompanied by similar reductions in peripheral skin temperature (~1–2 °C) and highlights an overall reduction in body/muscle temperature that may impact on performance [1]. Meckel et al. [10] reported that intense warm-up was required for better repeated sprint performance in young basketball players at the early stages of a match. Collectively the current and prior results highlight the importance of body temperature for prompt performance in basketball with substitutes unlikely to be optimally prepared.

Although CMJ wasn’t repeatedly tested throughout the inactivity period (mainly due to the act itself may have masked inactivity results) it was clear by the end of the first half the players performance was dramatically worse than post warm up and even pre warm up. This may be due to changes in arousal levels from the start of the warm up the end of the half.

However, overall the findings need to be treated with caution given the sample size (N = 2) limitation within this study. Future research should examine the best strategy and/or warm up to optimize basketball player preparation for competition. Additionally, further studies are needed to determine the impact of this inactive period on other important measures for successful competition such as reaction time, choice reaction time and decision making.

## 5. Practical Applications

- A standardized warm up considerably increases physiological and performance responses for basketball players that may contribute to enhanced match performance.
- Inactivity for a substantial period (6 min) following warm up demonstrates the players’ HR level returning to baseline and the start of skin temperature decrease obtained during the warm-up. Without running an off-court warm-up, substituting a player from this point on into the game may pose a risk for substitute strategies. However, the results of this study should be interpreted with caution given the small sample size.
- Implementation of a devised warm up to enhance core/skin temperature for substitute players immediately before they engage with competition may optimize player performances for a positive match outcome.
